# Treatment with Low-Dose Cytarabine in Elderly Patients (Age 70 Years or Older) with Acute Myeloid Leukemia: A Single Institution Experience

**DOI:** 10.4084/MJHID.2016.009

**Published:** 2016-01-01

**Authors:** Maël Heiblig, Mohamed Elhamri, Isabelle Tigaud, Adriana Plesa, Fiorenza Barraco, Hélène Labussière, Sophie Ducastelle, Mauricette Michallet, Franck Nicolini, Claudiu Plesa, Eric Wattel, Gilles Salles, Xavier Thomas

**Affiliations:** 1 Department of Hematology, Lyon-Sud Hospital, Hospices Civils de Lyon, Pierre Bénite, France; 2 Laboratory of Cytogenetics, Lyon-Sud Hospital, Hospices Civils de Lyon, Pierre Bénite, France; 3 Laboratory of Cytology and Immunology, Lyon-Sud Hospital, Hospices Civils de Lyon, Pierre Bénite, France; 4 Trarieux Clinic, Lyon, France.

## Abstract

**Objectives:**

Low-dose cytarabine (LD-AraC) is still regarded as the standard of care in elderly patients with acute myeloid leukemia (AML) ‘unfit’ for intensive chemotherapy. In this study, we reported our experience with LD-AraC in patients ≥ 70 years old and compared the results to those of intensive chemotherapy, best supportive care (BSC), or hypomethylating agents in the same age population.

**Methods:**

Between 2000 and 2014, 60 patients received LD-AraC at 20 mg once or twice daily by subcutaneous injection for 10 consecutive days every 4–6 weeks.

**Results:**

Complete remission rate with LD-AraC was 7% versus 56% with intensive chemotherapy and 21% with hypomethylating agents. Median overall survival (OS) of patients treated with LD-AraC was 9.6 months with 3-year OS of 12%. Survival with LD-AraC was better than with BSC only (*P* = 0.001). Although not statistically significant, intensive chemotherapy and hypomethylating agents tended to be better than LD-AraC in terms of OS (median: 12.4 months and 16.1 months, respectively). There was no clear evidence that a beneficial effect of LD-AraC was restricted to any particular subtype of patients, except for cytogenetics. There was a trend for a better OS in LD-AraC treated patients in the setting of clinical trials as compared with those treated outside of a clinical trial.

**Conclusions:**

Despite a trend in favor of intensive chemotherapy and hypomethylating agents over LD-AraC, no real significant advantage could be demonstrated, while LD-AraC showed a significant advantage comparatively to BSC. All this tends to confirm that LD-AraC can still represent a baseline against which new promising agents may be compared either alone or in combination.

## Introduction

Despite multiple advances in AML therapy, the treatment outcome for older patients with acute myeloid leukemia (AML) is unsatisfactory, especially for patients in their latter years. As people are living longer, the incidence of AML is increasing. The treatment outcome for patients aged 70 years or older has not improved significantly over the last two decades in spite of improved supportive care. Most of these patients do not receive intensive chemotherapy either because they decline or because they are not considered fit enough for such therapy. The basis on which patients are not considered fit enough for intensive chemotherapy remains not clear and varies considerably from one investigator to another. Clearly performance status remains an important factor in therapy planning. However, evaluation of ‘fitness’ remains unclear. Recent reports have shown that geriatric assessment methods, with a focus on cognitive and physical function, improve risk stratification and may inform interventions to improve outcomes for older AML patients.^1^ Others showed that candidacy for intensive therapy should be based on biological features of disease rather than on age.^2^

Although low-dose cytarabine (LD-AraC) has not been adopted universally, it still represents a treatment reference (at least in Europe) for patients considered ‘unfit’ for intensive chemotherapy. LD-AraC was investigated extensively more than 20 years ago. LD-AraC has been used in various schedules showing responses that included complete remission (CR).^3^–^9^ Its mechanism of action is still not completely clear, acting potentially through cytotoxic action and/or through induction of apoptosis by differentiation induction.^10^,^11^ LD-AraC is relatively well tolerated and can be given in an outpatient care setting. However, it can induce excess cytopenia although this may be a prerequisite for efficacy. In the literature, 10% to 20% of patients have been reported to achieve CR.^3^–^9^,^12^ Randomized studies between intensive and non-intensive treatments showed better responses in intensively treated patients, but no significant differences in terms of survival.^13^,^14^ LD-AraC has been demonstrated to be more beneficial than best supportive care and hydroxyurea among patients not fit for intensive therapy, although fitness was not defined for patients’ age > 70 years.^12^ LD-AraC therapy still represents a baseline against which novel drugs may be compared either alone or in addition to LD-AraC. Currently, the role of lower-intensity regimens is under active investigations.^12^,^15^–^19^ Recent randomized trials comparing DNA hypomethylating agents, azacitidine or decitabine, with LD-AraC found improved CR rates and better survival with hypomethylating agents.^15^,^19^

These studies urged us on analyzing our series of elderly patients treated with LD-AraC with the aim to evaluate whether this treatment could still represent a standard therapy in this patient population to which new treatments should be compared. We, therefore, evaluated the efficacy of LD-AraC, in a single institution experience, in patients aged 70 years or older, and compared it to that of other treatments received by patients of the same age (intensive chemotherapy, best supportive care (BSC), and lower intensity therapy based on hypomethylating agents).

## Patients and Methods

### Patients

In total, 234 patients (aged 70 years or older) with newly diagnosed AML have been seen in the Department of Hematology at Lyon-University Hospital from 2000 to 2014. From 2000 to 2006, patients with PS ≤ 2 were considered ‘fit’ by the local physician and received an intensive treatment approach systematically. No specific criteria for defining ‘fitness’ were used. After 2006, a more ‘personalized’ treatment using either intensive chemotherapy or lower-intensity therapies (including LD-AraC, decitabine or azacitidine) based on the clinical judgment of the treating physician and the availability of clinical trials were proposed.^20^ Most patients older than 70 years received, therefore, a non-intensive option. Any type of AML (de novo or secondary) was considered. Acute promyelocytic leukemia and blast transformation of chronic myeloid leukemia were excluded. Among the 234 patients (aged 70 years or older) with newly diagnosed AML, 60 patients (16%) received LD-AraC. They were compared to 85 patients treated with intensive therapy (anthracycline- and cytarabine-based chemotherapy), 34 patients treated with hypomethylating agents (12 by decitabine and 22 by azacitidine), and 43 patients receiving only BSC. The 12 remaining patients received other treatments in the setting of investigational trials and were not considered for the study.

### Treatment

On entry, patients received LD-AraC 20 mg once or twice daily (according to physician’s choice) by subcutaneous (sc) injection for 10 consecutive days. Subsequent courses of LD-AraC were administered at intervals of 4 to 6 weeks. Regarding the control groups, intensive chemotherapy consisted of a combination of intermediate-dose cytarabine with an anthracycline. Azacitidine was given at the dose of 75 mg/m^2^/day for 7 consecutive days by sc injection, and decitabine was administered by intravenous route once daily at 20 mg/m^2^ for 5 consecutive days. Subsequent courses of these low-intensity treatments were administered at intervals of 4 to 6 weeks until disease progression. All clinical trials received approval from the institutional review board and were conducted in accordance with the Declaration of Helsinki. All participants gave their written informed consent. Policies with regard to blood product support, antibiotic and anti-fungal prophylaxis, and treatment of febrile neutropenia were determined by established local practice. BSC consisted only in the application of these policies plus eventually the administration of hydroxyurea in order to control white blood cell (WBC) count in case of the proliferative disease. Patients receiving intensive chemotherapy were systematically hospitalized for induction chemotherapy (median hospitalization duration: 36 days) and consolidation chemotherapy courses. Blood product transfusions were systematically administered when hemoglobin was ≤ 80 g/l and platelets ≤ 20 × 10^9^/l. Requirements for transfusions were the same for patients treated with lower intensity therapies (LD-AraC or hypomethylating agents) while platelets were only transfused to patients with bleedings in the case of treatment by BSC alone. Hospitalization was reserved for patients with infectious complications or other severe complications for patients belonging to two last groups of treatment.

### Endpoints

CR was defined by bone marrow aspiration, which was required to consist of more than 50% normal cellularity with evidence of trilineage maturation and less than 5% bone marrow blasts, no evidence of extramedullary disease, and regeneration of the peripheral neutrophil count to 1.0 × 10^9^/l and the platelet count to 100 × 10^9^/l. The persistence of myelodysplastic features did not exclude the diagnosis of CR. Response to therapy was evaluated after one or two courses for patients treated with intensive chemotherapy, and after 4 to 6 courses for those treated by lower-intensity treatments. Overall survival (OS) was the primary endpoint. It defines the time from starting treatment to death from any cause. For remitters, disease-free survival (DFS) is the time from CR to first event (recurrence or death in CR).

### Statistical analyses

Surviving patients were censored at the end of September 2014 when follow-up was up to date for 95% of patients. Descriptive statistics was used to characterize patients and their disease. Categorical variables were compared between treatment options by Fischer exact tests. Continuous variables were analyzed by parametric tests (*t* tests) or nonparametric tests (Wilcoxon) as appropriate. Estimated probabilities of survival were calculated using the Kaplan-Meier method, and the log-rank test evaluated differences between survival distributions. All variables tested by univariate analyzes were included in the multivariate analysis. Multivariate analyzes used the Cox proportional hazard method for survival. Hazard ratios (HRs) with 95% confidence intervals (CIs) were calculated for the main endpoint. An HR < 1 indicated a benefit for one factor over another. All *P* values are two-tailed, with a P value ≤ 0.05 considered statistically significant. Computations were performed using BMDP PC-90 statistical program (BMDP Statistical Software, Los Angeles, CA, USA).

## Results

Between 2000 and 2014, 60 patients (aged 70 years or older) with newly diagnosed AML, including 35 de novo AML and 25 secondary AML, were treated in our Institution by LD-AraC. Characteristics and outcome of these patients were compared to those of patients treated during the same period of time by intensive chemotherapy (85 patients), hypomethylating agents (34 patients), or only BSC (43 patients). The characteristics of patients, split by the treatment type at onset, are provided in Table 1, which shows, as expected, differences according to the distinct therapeutic approaches.

The median number of treatment courses given was 5 for LD-AraC (range: 1 – 20^+^) with a median length of treatment of 5.1 months (range: 0.9 – 28^+^ months). The overall CR rate of patients treated with LD-AraC was 7% (4 of the 60 patients). The median number of courses to achieve CR was 4 (range, 3–9 courses). The CR rate was significantly better in patients treated by intensive chemotherapy (48/85 patients; 56%) (*P* < 0.0001) and in patients treated by hypomethylating agents (7/34 patients; 21%) (*P* = 0.09). Median OS of patients treated with LD-AraC was 9.6 months (95% CI, 5.8–13.5 months) with 3-year OS of 12%.

**Figure 1:**
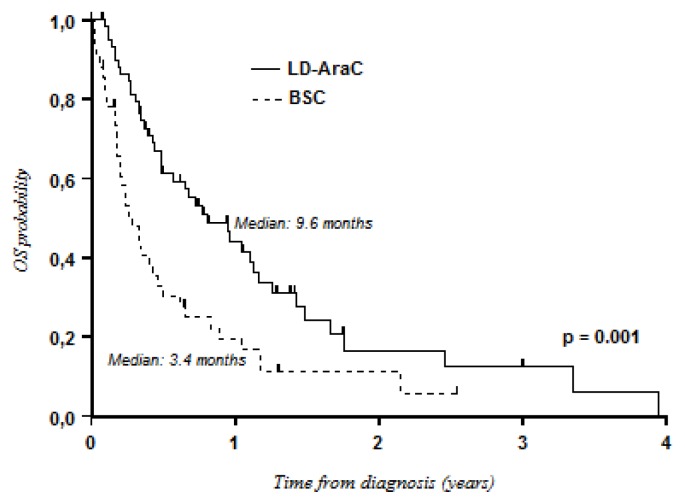
Overall survival: LD-AraC versus best supportive care.

**Figure 2:**
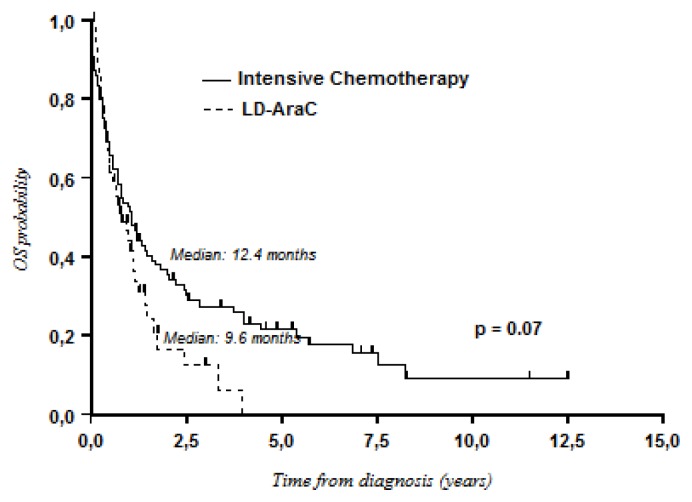
Overall survival: LD-AraC versus intensive chemotherapy.

**Figure 3:**
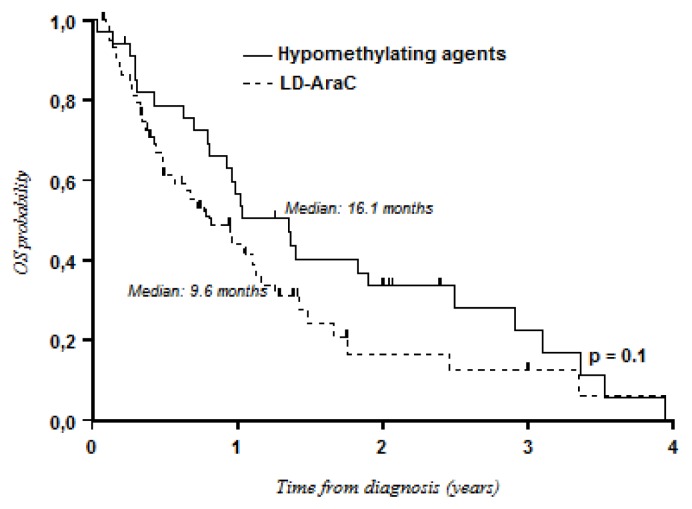
Overall survival: LD-AraC versus hypomethylating agents.

Survival with LD-AraC was better than that with BSC only (median OS: 9.6 months *vs.* 3.4 months; *P* = 0.001) (Figure 1). Although not statistically significant, intensive chemotherapy tended to be better than LD-AraC in terms of OS (median OS: 12.4 months *vs.* 9.6 months; 3-year OS: 27% *vs.* 12%; *P* = 0.07) (Figure 2). However, differences in favor of intensive chemotherapy were only confirmed for patients aged less than 75 years (median: 12.7 months *vs.* 9.2 months; 3-year OS: 28% *vs.* 10%). In patients aged ≥ 75 years, median OS was better with LD-AraC (9.6 months *vs.* 2.8 months). Although there was a trend for better results with hypomethylating agents, no significant differences were observed when compared with LD-AraC (median OS: 16.1 months with hypomethylating agents *vs.* 9.6 months with LD-AraC; 3-year OS: 22% *vs.* 12%; *P* = 0.1) (Figure 3). In a multivariate analysis including cytogenetics (unfavorable *vs.* intermediate/favorable risk), age (< 75 years *vs.* ≥ 75 years), de novo or secondary AML, and the type of treatment, only cytogenetics was of prognostic value (HR, 1.93; 95% CI, 1.50–2.47; *P* < 0.001).

There was no clear evidence that a beneficial effect of LD-AraC was restricted to any particular subtype of patients. In the univariate analysis, similar treatment effects were observed for all ages (< 75 years *vs* ≥ 75 years) (median OS: 9.2 months *vs* 9.6 months; *P* = 0.92), WHO PS (0–2 *vs* > 2) (median OS: 9.6 months *vs* 9.2 months; *P* = 0.63), bone marrow blastic infiltration at diagnosis (≤ 30% vs > 30%) (median OS: 17.7 months *vs* 9.2 months; *P* = 0.15), initial WBC count (≤ 10 × 10^9^/l *vs* > 10 × 10^9^/l) (median OS: 11.5 months *vs* 4.7 months; *P* = 0.35), and secondary AML (prior history of MDS or cancer *vs* no antecedents) (median OS: 5.8 months *vs* 13.5 months; *P* = 0.08). There was only a significant difference regarding initial cytogenetics (favorable and intermediate-risk *vs* unfavorable-risk) (median OS: 11.4 months *vs* 4.3 months; *P* = 0.03). In the multivariate analysis in a model taking into account all these factors, only initial cytogenetics and secondary AML appeared of prognostic value (Table 2).

Of the patients who received LD-AraC, 24 patients were treated inside clinical trials, while 36 patients were not. There were no substantial differences between those patients with respect to blood product support, hospitalization, and days on antibiotics. However, the clinical trials required significantly more day care visits for patients. Median OS was 13.2 months (95% CI, 8.6–15.1 months) for patients included in clinical trials *vs* 7.8 months (95% CI, 4.3–11.5 months) for those not included, with 3-year OS of 18% and 9%, respectively (*P* = 0.21) (Figure 4). There were no significant differences in terms of survival between patients receiving LD-AraC at 20 mg per day and those receiving 20 mg twice a day.

Most of the patients (92%) upon failure after LD-AraC therapy received only BSC with eventually a combination of 6-mercaptopurine with oral methotrexate. Five patients received a second line therapy: one with azacitidine and 4 with a new drug inside a phase 1 investigational trial.

## Discussion

Overall, despite a trend in favor of intensive chemotherapy and hypomethylating agents over LD-AraC, no real significant advantage could be demonstrated in terms of OS, while LD-AraC showed a significant advantage comparatively to BSC. CR rates were higher with intensive chemotherapy or treatment by hypomethylating agents than with LD-AraC, but this did not translate into a significant benefit in terms of OS. The old principle of achieving a CR with intensive chemotherapy to convey a favorable outcome might not apply to this patient population, for which OS and quality of life represent the most relevant endpoints. However, one recent study regarding the quality of life beyond 6 months after diagnosis in this patient population showed that achievement of CR is associated with improvements in global health, physical function, and role function without negatively affecting other health domains.^21^ In our series, the prolonged OS contrasting with the low CR rate after treatment by LD-AraC could be explained by the pursuit of treatment as long as the disease was controlled and that the treatment was considered beneficial for the patient and the selection of patients with the orientation of frailer patients directly to BSC alone. This approach explained the higher median number of treatment courses given in our study comparatively to the median number of courses given in previous studies.^15^,^19^,^22^ The same therapeutic behavior applied to hypomethylating agents can also explain the differences between our findings and the recently published studies of decitabine and azacitidine in elderly AML patients.^15^,^19^

Our results with LD-AraC showed lower CR rates but a median OS better than those observed in previous studies.^4^,^12^,^23^ Differences in terms of CR rates could be explained by the different schedules used. The response to LD-AraC appears dose dependent. Burnett et al., who reported 18% to 21% of CR rate, used AraC at 20 mg twice daily for 10 consecutive days,^12^,^22^ while Rodriguez and Tilly, who observed 28%^2^ to 32%^4^ of CR, used LD-AraC for a longer period of time (cycles of 21 days). Theoretically, patients who achieved CR have a better median survival compared with those who did not achieve CR. However, higher doses or longer duration schedules are associated with a longer period of hypoplasia. Actually our better results in terms of median OS could be explained by a higher rate of severe toxicity in schedules with higher doses and longer treatment^4^,^23^ and also by recent improvements in terms of supportive care.^20^ Supportive care improvements over the last decade were also evidenced by the difference in outcome between our series and that published by the M.D. Anderson Cancer Center few years ago regarding patients aged ≥ 70 years receiving intensive chemotherapy.^24^ They reported about patients treated between 1990 and 2008 and found 45% of CR and a median OS of only 4.6 months. This datum is close to the results we previously reported during the same period of time,^20^ while our current series of patients treated with intensive chemotherapy showed a significant improvement in higher CR rates and longer survival, in relationship with improvements in supportive care. An important point from our study was a tendency for better OS in patients treated inside clinical trials comparatively to those who were not. This datum stresses one more time on the importance of a regular follow-up and on supportive care in this patient population, and can explain the better survival with LD-AraC in our series as compared to previous ones.^4^,^12^,^23^

The strength of our study is that this is a report of treatment with LD-AraC involving only elderly AML patients aged ≥ 70 years and, therefore, reporting on a relatively homogeneous cohort of patients. Our study, however, suffers from limitations. As expected, patient characteristics varied significantly among the four groups of treatment. Given the nature of clinical practice, it is conceivable that relatively fit patients were treated with intensive chemotherapy while less fit were offered LD-AraC or hypomethylating agents, and frail patients received only BSC. Other limitations mainly concerned the retrospective profile of the study with unbalanced distribution of the treatment options, the small size of the cohorts, the absence of data regarding comorbidities (such as diabetes, high blood pressure, or cardiac pathology), absence of any quality of life questionnaire, and the under-representation of patients who received intensive chemotherapy during the last period of study while the hypomethylation cohort belongs mainly to this same recent period. The main goal of our study was to report the results of LD-AraC therapy in the real life of one hematology center. In this setting, comparisons among treatments used in this patient population were authorized, although involving unbalanced groups.

Although a benefit in OS has been demonstrated with LD-AraC compared with BSC, outcome with LD-AraC remains unsatisfactory. A true step forward in the treatment of AML in elderly patients can be expected from the development of more effective therapies and the further improvement of supportive measures. Recently, a lower-intensity, prolonged-therapy program testing clofarabine and LD-AraC alternating with decitabine was well tolerated and highly effective in older patients with AML.^25^ Hypomethylating agents also represent a promising alternative to intensive chemotherapy in this patient population.^15^,^19^ On the basis of our findings, LD-AraC did not show sufficient evidence of benefit over hypomethylating agents to be considered again as the standard treatment for this patient population, but can still represent a baseline against which new promising agents may be compared either alone or in combination.^22^

## Figures and Tables

**Table 1:** Patient characteristics and outcome split by initial treatment type.

*Characteristics*	LD-AraC (60 patients)	Intensive chemotherapy (85 patients)	Hypomethylating agents (34 patients)	Best supportive care (43 patients)

Age (years)	76 (70–84)^a^	72 (70–79)	76 (70–86)	76 (71–89)

Gender (M/F)	39/21	45/40	20/14	27/16

WHO PS > 2	4 (7%)	1 (1%)	0	10 (23%)

FAB criteria:				
M0/M1/M2/M4	4/10/21/8	2/19/24/8	2/2/12/3	3/5/14/4
M5/M6/M7/ND	8/1/0/8	27/1/3/1	4/4/1/6	5/1/0/11

Antecedents:				
Oncology	18 (30%)^b^	14 (16%)	9 (26%)	11 (26%)
MDS	8 (13%)	20 (24%)	10 (29%)	9 (21%)

Cytogenetics:^c^				
Favorable	1 (2%)	2 (2%)	0	1 (2%)
Intermediate	38 (63%)	48 (56%)	15 (44%)	9 (21%)
Unfavorable	13 (22%)	16 (19%)	16 (47%)	16 (37%)
Failure	8 (13%)	19 (23%)	3 (9%)	17 (40%)

LDH (UI/l)	317 (149–2998)	466 (124–2909)	394 (164–17773)	412 (149–6825)

Peripheral blood:				
Hb (g/l)	97 (53–136)	93 (62–152)	93 (39–126)	83 (41–131)
WBC (×10^9^/l)	4.4 (0.4–117.9)	3.4 (0.5–223)	2.2 (0.3–45.3)	2.3 (0.6–62.3)
Platelets (×10^9^/l)	75 (5–261)	52 (6–285)	60 (15–214)	66 (3–598)
PMN (%)	24 (1–67)	15 (0–97)	26 (1–67)	19 (0–72)
Blasts (%)	12 (0–90)	15 (0–97)	3 (0–63)	10 (0–95)

Bone Marrow:				
Blasts (%)	50 (20–95)	62 (20–100)	30 (20–90)	53 (20–95)
Outcome:				
CR	4/60 (7%)	48/85 (56%)	7/34 (21%)	1/43 (2%)
OS (median)	9.6 months	12.4 months	16.1 months	3.4 months

a Median (range);

b Number of cases (percentage);

c The favorable risk category included patients with inv(16)/t(16;16)/del(16q), or t(8;21), with or without other chromosome abnormalities; the intermediate risk category included patients characterized by +8, −Y, +6, del(9q), del(12p) or normal karyotype; the unfavorable risk category was defined by the presence of one or more of −5/del(5q), −7/del(7q), inv(3q)/t(3;3), abnormal 20q or 21q, translocation involving 11q23, t(6;9), t(9;22), abnormal 17p or complex karyotype, defined as 3 or more chromosomal abnormalities.

Abbreviations: Hb, hemoglobin; LDH, lactate dehydrogenase; MDS, myelodysplastic syndromes; M/F, male/female; ND, not determined; PMN, polymorphonuclear; WBC, white blood cell; WHO PS, World Health Organization performance status.

**Table 2:** Multivariate analysis: Factors associated with overall survival in patients treated with LD-AraC.

*Factor*	HR	95% CI	*P* value
**Initial cytogenetics** (Unfavorable *vs* Favorable/Intermediate)	1.95	1.05 – 4.03	0.04
**Secondary AML** (no antecedents *vs* prior malignant history)	0.46	0.22 – 0.96	0.05

Age, WHO performance status, bone marrow blast cells, cytogenetics, secondary AML, WBC count were factors included in the model. A HR < 1 indicated a benefit for one factor over another.
